# Insoluble-Bound Phenolics in Food

**DOI:** 10.3390/molecules21091216

**Published:** 2016-09-10

**Authors:** Fereidoon Shahidi, JuDong Yeo

**Affiliations:** Department of Biochemistry, Memorial University of Newfoundland, St. John’s, NL A1B 3X9, Canada; judy1894@naver.com

**Keywords:** bound phenolics, localization and transfer of phenolics, bioactivity, release from cell wall matrix

## Abstract

This contribution provides a review of the topic of insoluble-bound phenolics, especially their localization, synthesis, transfer and formation in plant cells, as well as their metabolism in the human digestive system and corresponding bioactivities. In addition, their release from the food matrix during food processing and extraction methods are discussed. The synthesis of phenolics takes place mainly at the endoplasmic reticulum and they are then transferred to each organ through transport proteins such as the ATP-binding cassette (ABC) and multidrug and toxic compound extrusion (MATE) transporter at the organ’s compartment membrane or via transport vesicles such as cytoplasmic and Golgi vesicles, leading to the formation of soluble and insoluble-bound phenolics at the vacuole and cell wall matrix, respectively. This part has not been adequately discussed in the food science literature, especially regarding the synthesis site and their transfer at the cellular level, thus this contribution provides valuable information to the involved scientists. The bound phenolics cannot be absorbed at the small intestine as the soluble phenolics do (5%–10%), thus passing into the large intestine and undergoing fermentation by a number of microorganisms, partially released from cell wall matrix of foods. Bound phenolics such as phenolic acids and flavonoids display strong bioactivities such as anticancer, anti-inflammation and cardiovascular disease ameliorating effects. They can be extracted by several methods such as acid, alkali and enzymatic hydrolysis to quantify their contents in foods. In addition, they can also be released from the cell wall matrix during food processing procedures such as fermentation, germination, roasting, extrusion cooking and boiling. This review provides critical information for better understanding the insoluble-bound phenolics in food and fills an existing gap in the literature.

## 1. Introduction

Phenolic compounds play a significant role in plants by regulating growth as an internal physiological regulator or a chemical messenger [1]. They also affect the growth hormone auxin (IAA) in which monohydroxy B-ring flavonoids act as a cofactor of peroxidase, causing degradation of IAA while dihydroxy B-ring flavonoids inhibit the degradation process [2,3]. In addition, quercetin, apigenin and kaempferol can bind to plasma membrane protein (receptor), thereby interfering with the transport of polar auxin compounds through the membrane, thus affecting plant architecture and growth [1].

Phenolic compounds are also responsible for the protection of plants from sunlight by absorbing harmful short high energy wavelengths at the electron rich parts such as π-bond of the aromatic rings, leading to reduced oxidative stress. They can also protect the plants from exterior predators such as insect with their possible toxic effects or protein precipitation and hence a puckering effects in the mouth as well as playing a role in symbiosis and serving as attractant for pollinators, among others.

Phenolics can be classified into several groups such as phenolic acids, flavonoids, stilbenes, coumarins, lignins and tannins. Phenolic acids include hydroxybenzoic acids (C_6_–C_1_) such as gallic, *p*-hydroxybenzoic, vanillic, syringic, protocatechuic and ellagic acids as well as hydroxycinnamic acids (C_6_–C_3_) such as *p*-coumaric, caffeic, ferulic, sinapic and chlorogenic acids. The flavonoids are composed of a three ring structure in the C_6_–C_3_–C_6_ form, which constitute different classes of compounds such as flavones, flavanonse, flavonols, flavanonols, isoflavones, flavanols and anthocyanidins, based on different substitution patterns such as hydroxyl and methoxy groups. Stilbenes are also one group of phenolics that includes resveratrol. Coumarins, as a benzopyrone group are also present in different foods. Moreover, phenolics are also present in the polymeric form in plants; lignins are the polymer of monolignols such as *p*-coumaric acid and sinapic acid. While tannins are polymeric phenolics. The tannins exist as hydrolyzable tannins such as ellagitannins and as condensed tannins, also known as a proanthocyanidin which are polymers of catechin and epicatechin. The latter group are further divided into several groups such as A, B and C, depending on the type of linkage between the flavonoids, namely single or double linkages.

The phenolic compounds have attracted great interest from related fields due to their effective antioxidant capacity in both in vitro and in vivo studies. Phenolic compounds can be divided into free, esterified and insoluble-bound forms, depending on whether they occur in the free form or are covalently bound to other molecules such as fatty acids (soluble esters) or insoluble macromolecules (insoluble-bound phenolics). Most insoluble-bound phenolics chemically form covalent bonds with cell wall substances including pectin, cellulose, arabionoxylan and structural proteins and account for relatively large amount (20%–60% in vegetable, fruits and legume/seeds) compared to the soluble phenolics in foods [4]. Given the currently available analytical methods for the measurement of the contents of insoluble-bound phenolics, perhaps higher than expected amounts of insoluble-bound phenolics are present in natural sources.

In this review, we discuss the insoluble-bound phenolics by focusing on their localization, biosynthesis, transfer and forming of covalent bonds in the cell walls. Subsequently, their content in natural food sources, their metabolism in the human digestive track and bioactivities, as well as extraction methods and release from cell wall matrix during food processing are discussed.

## 2. Results

### 2.1. Localization of Soluble and Insoluble-Bound Phenolics

**Figure 1:**
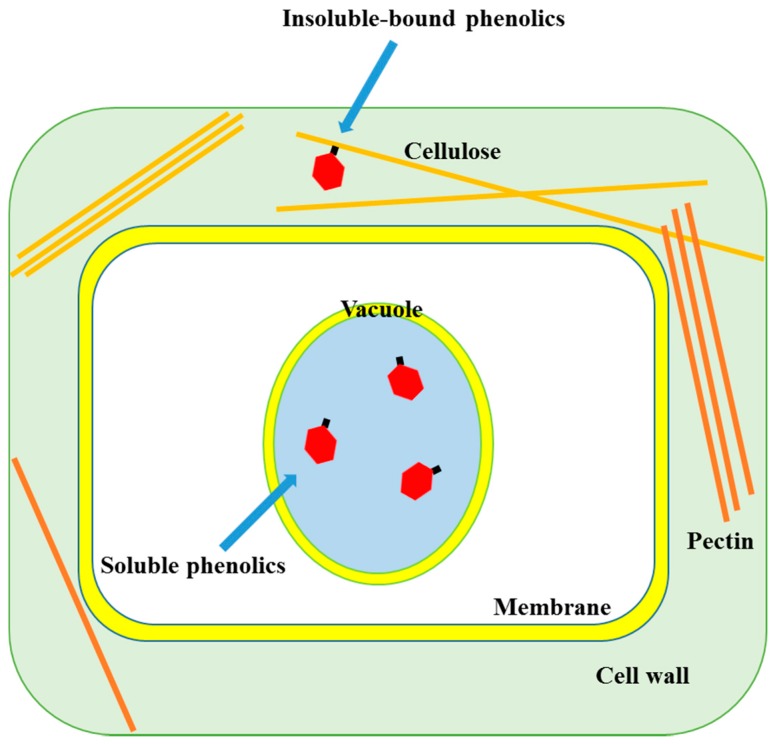
Localization of soluble and insoluble-bound phenolics in plant cell (other organs are not shown).

**Figure 2:**
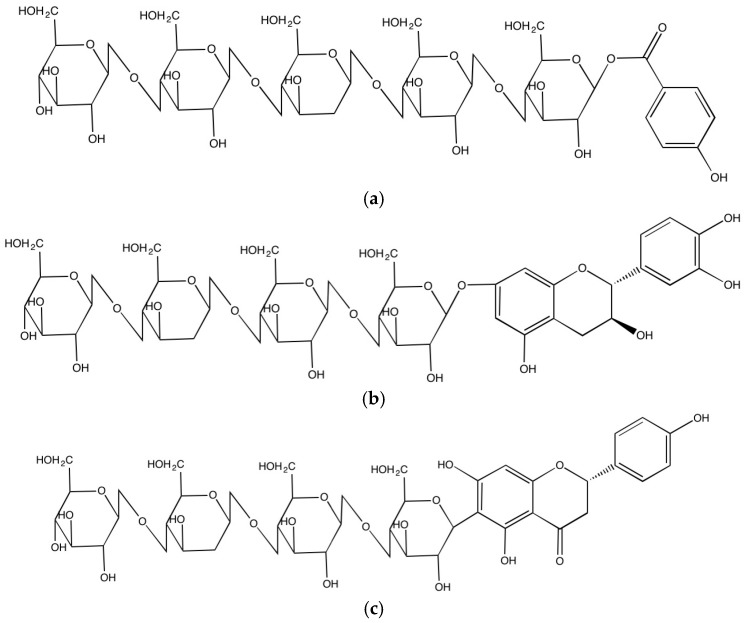
Representative covalent bonds found in insoluble-bound phenolics; (**a**) ester bond of 4-hydroxy benzoic acid (**b**) ether bond of catechin and (**c**) carbon-carbon bond of naringenin attached to cellulose molecule.

Phenolic compounds occur in both soluble and insoluble-bound forms. Most of the soluble phenolics are localized in the vacuoles of plant cells where they are trapped (Figure 1). When phenolics are incorporated inside a vacuole, the low pH induced by the high concentration of organic acids inside the vacuole leads to their localization [5]. On the other hand, insoluble-bound phenolics are localized in cell wall matrix of the plant cells. The phenolic compounds synthesized in the intracellular organs, mainly the endoplasmic reticulum, are released and transported into the vacuole or cell wall matrix through the vesicle transfer system that is a small lipid bilayer system, which can contain phenolics and facilitates their migration into the cell wall matrix [6]. The transported phenolic compounds are bound to macromolecules such as structural proteins, cellulose and pectin through covalent bonds via ether, ester and carbon-carbon bonds in the cell wall matrix, forming insoluble-bound phenolics (Figure 2). Thus, most of the soluble and insoluble-bound phenolics are localized in the intracellular organs/sites of plant cells.

Seeds/legumes consist of the outer part, known as seed coat/hull and inner part, including endosperm, epicotyl, hypocotyl and radicle. The endosperm of legumes accounts for a considerable weight of the seeds, acts as a nutrition storage organ and is consumed during the germination process. Most nutritional compounds of endosperms are starch, protein and lipid, accounting for 7%, 12% and 5% of their total dry weight, respectively [7]. Thus, most of the endosperm consists of storage cells, implying a low content of phenolic-containing cells. On the other hand, the seed coat/hull is composed of epidermis, hypodermis, chlorenchyma, palisade, parenchyma and endothelium cells, all of which contain most of the organs such as vacuoles and cell walls, thus containing high amounts of phenolics in both the soluble and insoluble-bound forms.

Leaves and stems, as well as other essential parts of plants, also possess phenolic-containing cells such as epidermal cells, guard cells, subsidiary cells and epidermal hairs (trichomes) in the leaf and epidermal cells, parenchyma cell, chlorenchyma cell and collenchyma cell in the stem [8]. Therefore, phenolics are ubiquitous secondary plant metabolites of natural sources especially in the cell concentrated portion of plants.

### 2.2. Synthesis, Transport and Formation of Insoluble-Bound Phenolics

#### 2.2.1. Synthesis

The synthesis of phenolic compounds takes place mostly at the cytoplasmic surface of endoplasmic reticulum, which is continuous with the outer nucleus of the plant cell. Many types of enzymes are involved in transforming the structure of phenolics, leading to different classes, namely phenolic acids, flavonoids, including anthocyanidins and other types of phenolics.

Phenylalanine, one of essential amino acids synthesized through the shikimate pathway of erythrose-4-phosphate and phosphoenolpyruvate, is a precursor for the synthesis of phenolic compounds and also tyrosine, albeit to a lesser extent. Phenylalanine releases the ammonia group via the action of phenylalanine ammonia lyase (PAL), yielding *trans*-cinnamic acid with the formation of a double bond. The *trans*-cinnamic acid is transformed into *p*-coumaric acid by the action of P450 monooxygenase, followed by caffeic acid with hydroxylase and then, ferulic acid by *o*-methyl transferase. Ferulic acid is further altered into sinapic acid by hydroxylase and *o*-methyl transferase to add a methoxy group to the molecule. Benzoic acid derivatives can be formed by the loss of a two carbon atom moiety from phenylpropanoids.

Flavonoids’ synthesis, known as the flavonoid branch pathway, is initiated by combining *p*-coumaroyl CoA and three molecules of malonyl CoA, converting them into naringenin chalcone by the action of chalcone synthase (CHS), followed by flavonone through chalcone isomerase. The resultant flavonone may be converted into different types of flavonoids such as flavononol, flavonol, isoflavone, flavone, anthocyanins and catechins via individual enzymatic reactions. For example, genistein, kaempferol and apigenin can be synthesized by isoflavone synthase, flavonol synthase/flavanone-3-hydroxylase and flavone synthase, respectively, from the flavonone.

#### 2.2.2. Transport

**Figure 3:**
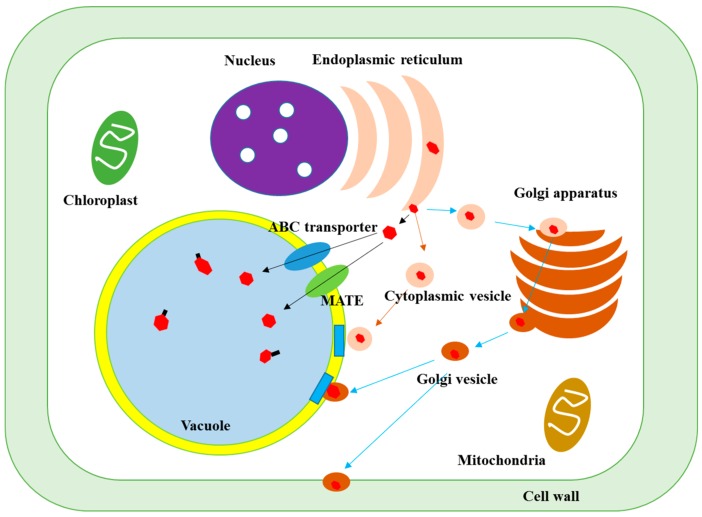
Transfer mechanisms of phenolics after synthesis at the endoplasmic reticulum in plant cell. (ABC: ATP-binding cassette; MATE: multidrug and toxic compound extrusion).

The synthesized phenolic compounds in the cytoplasmic surface of endoplasmic reticulum are transferred to other organs of plant cells through vesicle transfer system and membrane- mediated transport [9,10,11,12], as shown in Figure 3.

The first possible transfer mechanism is the membrane-mediated transport. After synthesis at the endoplasmic reticulum, phenolics are released to the cytoplasmic space and can enter to the organs inside by penetrating the compartment membrane through the transporter protein [13]. For example, the ATP-binding cassette (ABC) transporter and multidrug and toxic compound extrusion (MATE) proteins are the main route for the incorporation of glucosides of anthocyanins, apigenin and catechins to the vacuole tonoplast [14].

Another transfer mechanism is via the cytoplasmic vesicle. The phenolics synthesized are secreted into the cytoplasmic space and are incorporated by a lipid bilayer membrane, called cytoplasmic vesicle or sac, followed by their transfer to each cell organ such as vacuoles, nucleus and cell wall, which is known as a vesicle transfer system, facilitating the delivery of phenolics to the target site by protecting them from other reactive compounds and enzymes present in plant cells. When the cytoplasmic vesicles arrive at the surface of organs, they release their phenolic contents to the organ inside by fusing to their membrane [10].

In addition, the cytoplasmic vesicles can also be absorbed into the Golgi apparatus, leading to transformation of the phenolic structures due to the presence of multiple enzymes, followed by their release to the cytoplasmic space by wrapping inside of Golgi vesicles that transfers the phenolics to each organ.

#### 2.2.3. Formation of Insoluble-Bound Phenolics in the Cell Walls

The exact mechanism for the transfer of phenolics to the cell wall and formation of insoluble- bound phenolics has not yet been well established. However, there are some evidences about the transfer of phenolics to the cell wall matrix [12,15,16]. The cytoplasmic and Golgi vesicles containing phenolics can move to the plasma membrane and secrete the phenolics to the cell wall matrix [6]. In addition, the phenolics in the cytoplasmic can be transported to the plasma membrane through ABC transporters and across the plasma membrane, reaching the cell wall matrix [12].

In the cell wall, the released phenolic compounds can form covalent bonds with cell wall substances such as cellulose, hemicellulose, arabinoxylans, structural proteins and pectin through ester, ether and C-C bonds. The carboxyl group of phenolic acids such as benzoic and cinnamic acids can form ester bonds with hydroxyl groups of cell wall substances [17] and can also yield ether bonds by binding between hydroxyl groups of phenolic compounds and hydroxyl groups of cell wall substances and C-C bonds as well when they directly create covalent bond between carbon atom of phenolics and carbon atom of cell wall substances. The covalent bonds so formed play a major role in connecting cell wall substances, enhancing the rigid structure of the cell wall matrix. In addition, cell wall phenolics protect them from a number of pathogens and penetration of fungi as well as UV damage [18,19].

### 2.3. Phenolic Acids and Flavonoids Contents in Cereals, Legumes and Other Seeds

#### 2.3.1. Phenolic Acids

Phenolic acids are the main bound compounds in natural sources such as cereal grains, legumes and other seeds, acting as building materials for the cell wall matrix by connecting between insoluble macromolecules such as cellulose, hemicellulose and pectin, supporting the formation of rigid cell wall structures. Cinnamic acids and benzoic acids are representative groups of phenolic acids. Cinammic acids include *p*-coumaric, caffeic, ferulic and sinapic acids, while benzoic acids encompass *p*-hydroxybenzoic, protocatechuic, vanillic, syringic and gallic acids. The differentiation in substituted functional groups such as hydrogen, hydroxyl and methoxy groups dictate their molecular structures, influencing their antioxidant capacity and bioactivity.

Recently, a variety of insoluble-bound phenolics and their contents in different plant sources have been reported, as shown in Table 1. In cereals, bound phenolic acids such as protocatechuic, 4-hydroxybenzoic, vanillic, *p*-coumaric and ferulic acid were found in black rice and their contents were 162.1, 21.2, 27.7, 17.6 and 64.7 μg/g, respectively [20]. Das and Singh [21] reported that maize possessed bound phenolic acids such as *p*-hydroxybenzoic, vanillic, syringic, caffeic, *p*-coumaric, ferulic and isoferulic acid and their contents depended on the part of maize grain, thus pericarp portion was more abundant in bound phenolics than the germ and endosperm. Aside from the above investigations, a number of studies have been conducted on the identification and quantification of bound phenolics acids in cereals such as durum wheat, bread wheat, barley, oat, rye, rice, triticale and millet, revealing similar bound phenolic composition with cereals discussed above [22,23,24].

The bound phenolic acids are also abundant in legumes. Alshikh et al. [24] studied six lentil cultivars and reported the composition and contents of bound phenolic acids such as gallic (0.0–1.8 μg/g), protocatechuic (0.0–4.4 μg/g), *p*-coumaric (0.0–3.1 μg/g) and ferulic acid (0.0–0.3 μg/g). Chen et al. [25] reported that cranberry beans contained *p*-hydroxybenzoic, *p*-coumaric, ferulic and sinapic acid in the bound form and their contents were 4.4–71.2, 4.9–18.4, 11.0–41.7 and 0.03–3.55 μg/g of whole bean in seven different cultivars, respectively. Other legumes such as mung bean, pinto bean, black bean, kidney bean, cow bean, chick pea were also rich in bound phenolic acids as shown in Table 1. In addition, protocatechuic acids were found in cow gram, flower waist bean, hyacinth bean and pearl bean as the main bound phenolic acids, ranged from 110 to 172 μg/g of DW [30].

Bound phenolic acids are also present in oilseeds. Sunflower seeds contain gallic, protocatechuic, caffeic, *p*-coumaric, ferulic and sinapic acids and their contents were ranged from 2.5 to 50.8 μg/g of DW [27]. Naczk and Shahidi [31] investigated the bound phenolic acids in rapeseed meal and found sinapic, *p*-coumaric and trans-ferulic acids as the main insoluble-bound phenolics. Aside from major oilseeds, flaxseed, moringa oleifera seed flour and soybean were also studied for their bound phenolic acids and showed high contents of gallic and *p*-coumaric acids as shown in the Table 1.

According to the literatures, fruit seeds were also valuable sources for bound phenolic acids such as protocatechuic, *p*-coumaric, gallic, caffeic and syringic acids. Those of phenolic acids were found in blackberry, black raspberry, blueberry seed meals and the range of individual phenolics acids were 39–221, 25–243, 242–356, 25–217 and 0–93 μg/100 g, respectively [35].

#### 2.3.2. Flavonoids

Flavonoids, as plant secondary metabolites, are abundant in natural sources such as legumes, cereals and other seeds. They consist of three ring structure in the C_6_–C_3_–C_6_ form, with different substitutions such as hydroxyl and methoxy groups, which constitute different classes of flavonoids such as flavonols, flavanols and flavone as well as proanthocyanidins by forming oligomers and polymers. In the plant life, flavonoids perform various roles such as UV-light protection, defense from external predators such as insects and controlling auxin transport [9]. They may be present in both of the soluble and insoluble-bound forms. Different bound flavonoids have been reported in the literature and these are summarized in Table 2.

Cereals are the main natural sources of bound flavonoids. Das and Singh [21] investigated bound flavonoids in maize and they found 15.9 μg/g of kaempferol in pericarp portion and 28.8 μg/g of quercetin in germ parts, but the bound flavonoids were not detected in the endosperm portion. In addition, three major flavonoids, namely catechin (16.28 μg/g), quercetin (47.2 μg/g) and kaempferol (30.4 μg/g) were identified in quinoa seeds [36]. Other cereals such as rice, corn, wheat and barely were also great sources of bound flavonoids, showing 240, 760, 430 and 370 μg catechin equivalents/g of DW, respectively, however their compositions were not provided [34].

Aside from cereals, legumes are also valuable sources of bound flavonoids. For example, several bound flavonoids such as catechin (15.0–78.4 μg/g), epicatechin (0.5–7.9 μg/g), (+)-catechin-3-glucoside (0.0–122.0 μg/g) and luteolin 3′-7-diglucoside (0.0–49.3 μg/g) were found in six lentil cultivars [24]. In addition, cranberry beans contained bound flavonoids such as kaempferol (0.27–0.37 μg/g) in four different cultivars [25]. Other legumes such as black bean, cow gram, hyacinth bean, pearl bean, red bean, red kidney bean and spring bay bean were also rich in bound flavonoids, with quercetin and catechin being the most prevalent flavonoids in the aforementioned legumes [30].

Oilseeds and other seeds are also abundant in bound flavonoids. For example, *Moringa oleifera* seed flour possessed three major flavonoids, namely catechin (7490 μg/g), epicatechin (810 μg/g) and quercetin (18.7 μg/g) [33]. Sunflower seeds contained quercetin, kaempferol and apigenin at 1.5, 0.5 and 2.9 μg/g, respectively. In addition, epicatechin, isoquercitrin and quercetin were found in soybean in high levels as shown in Table 1 [30]. The concentration of bound flavonoids were largely dependent on the food sources and analytical methods employed in the hydrolysis and extraction procedures. In addition, fruitseeds were also a rich source of bound flavonoids. Ayoub et al. [35] reported that bound flavonoids such as (+)-catechin, (−)-epicatechin, quercetin, epigallocatechin, myricetin, quercetin pentose, epicatechin gallate, kaempferol hexoside, quercetin-3-O-glucuronide were present in blackberry, black raspberry, blueberry seed meals and their contents were 40–102, 7–116, 27–326, 40–136, 0–26, 0–330, 0–117, 0–68 and 0–1149 μg/100 g, respectively.

### 2.4. Metabolism of Insoluble-Bound Phenolics in Human Digestive Tract

Foods containing phenolic compounds undergo multi-enzyme reactions, following alteration of physical and chemical properties in the digestive tract, including the mouth, stomach, as well as small and large intestines (colon) after intake. Phenolic compounds can be released from the food matrix in the gastrointestinal tract by enzymes and pH conditions [38]. The released free phenolics are absorbed in the small intestine, followed by conjugation with other compounds, leading to their introduction in the blood circulation system [39]. However, only 5%–10% of phenolics can be absorbed in the small intestine and the remaining 90%–95% move directly to the colon. Given the argument of Rodríguez-Roque et al. [40] who stated that the phenolic bioavailability can be defined as the amount of phenolic compounds that reaches the blood circulation system so that they can exert bioactivity at the tissue and cells, 5%–10% absorption rate of phenolics indicates their low bioavailability. Conversely, insoluble-bound phenolics are not absorbed in the small intestine because they are bound to insoluble macromolecules such as cellulose, hemicellulose, structural protein and pectin. Thus, they reach the colon (large intestine), where they undergo fermentation by the colon microbiota and release the bound phenolics [41,42]. In the colon, a variety of microorganisms, approximately 1000 different bacteria and around 10^14^ colony forming units (CFU), exist and take part in the fermentation of unabsorbed material as well as fungi, protozoa and archaea [43]. The microorganisms, including *Bifidobacterium spp.* and *Lactobacillus spp.* secrete a variety of extracellular enzymes such as carbohydrases, proteases and other types of enzymes, leading to the disruption of cell wall matrix or hydrolysis of covalent bonds of bound phenolics, followed by liberation of phenolics. The released phenolics render a myriad of health benefits that influence the fermentation environment of colon by decreasing pH and preventing the growth of cancer-inducing microorganisms. Yi et al. [44] reported that phenolic compounds from blueberry inhibited proliferation of colon cancer cell line such as HT-29 and Caco-2 by approximately 50%.

During fermentation in the colon, many different types of products are formed, however only a few of them can be absorbed. The main compounds that can be absorbed in the colon include water and some salts and their sodium and potassium ions. In addition, vitamin K, vitamin B12, thiamin, riboflavin and short fatty acids can also be absorbed. However, absorption of phenolics has not yet been well established, thus additional research should be carried out on the absorption of phenolics in the human colon to support the above bioactivities such as inhibitory activity of colon cancer.

### 2.5. Bioactivities of Insoluble-Bound Phenolics

#### 2.5.1. Phenolic Acids

In recent years, a variety of bioactivities of phenolic acids have been reported. First, gallic acid has shown great anti-cancer activity in a number of studies [45,46,47,48]. Gallic acid (GA) and methyl gallate (MG) extracted from *Givotia rottleriformis* reduced the growth of human epidermoid carcinoma (A431) skin cancer cells [49] and they exhibited an inhibitory activity against hepatitis C virus (HCV), which is a major blood-borne pathogen and causes liver cirrhosis and hepatocellular carcinoma (HCC) that is an infection in primary human hepatocytes [50,51]. In addition, methyl gallate possesses anti-bacterial and anti-viral properties [52,53,54]. Ferulic acid, which has been used to relieve angina pectoris and hypertension in China [55], showed anti-cancer activity in cultured MIA PaCa-2 human pancreatic cancer cell [56]. Ferulic acid was also effective against diabetes, cardiovascular disease, neurodegenerative disease and cancer [57,58,59]. Anti-cancer studies of ferulic acid were tested in different cell line systems such as those of breast cancer [58,60] colon cancer [61] and pancreatic cancer [58]. Feruloyl-L-arabinose was also effective when used on H1299 lung cancer cells [62]. Coumaric and caffeic acids revealed anticancer activity in human lung (A549) and colon adenocarcinoma (HT29-D4) cancer cell lines [63].

#### 2.5.2. Flavonoids

Flavonoids possess effective antioxidant capacity due to the presence of functional groups such as hydroxyl groups, which render bioactivities such as anticancer, anti-inflammation and ant-virus as well as reducing cardiovascular diseases, type-2-diabetes and cholesterol. A variety of bioactivities of bound flavonoids have been demonstrated. Lin et al. [64] studied the bioactivity of (+)-epigallocatechin 3-*O*-gallate extracted from roots of *Limonium sinense* and showed suppression of virus type-1 infection. Troxerutin, a derivative of rutin, showed a strong interaction and affinity for the major or minor groove of DNA structure of cancer cell, leading to the potential for killing cancer cells by radiation induced sensitizing, namely acting as a chemopreventive agent [65]. Apigenin exhibited radiation-induced sensitizing effect on lung carcinoma cells [66]; rhamnetin and cirsiliol were also showed the same effectiveness in tumor cells [67]. Flavonoids such as hesperidin, rutin, quercetin and naringin abated inflammation, NF-kB activation and apoptosis, which was caused by cisplatin (anticancer drug) [68,69,70,71]. Apigenin also attenuated cisplatin-induced nephrotoxicity in human renal proximal tubular epithelial cells [72]. Quercetin has been well documented as a great functional compound for the prevention of cancer, cardiovascular diseases and cognitive malfunction [73,74,75]. Kaempferol revealed anticancer activity in human lung cancer cells and in vitro cancer cell line systems [76,77]. In addition, kaempferol suppressed other human cancer cell lines such as human cervical carcinoma (Hela), human stomach carcinoma (SGC-7901), human lung carcinoma (A549) and human breast carcinoma (MCF-7) by inducing nuclear condensation and mitochondria dysfunction [78]. Other bioactivities such as anti-inflammation, analgesic, antiallergic, cardioprotective, antidiabetic and osteoporotic activities were also found for kaempferol [79,80,81,82]. Myricetin inhibited cancer cells in rat and colon carcinogenesis [83,84]. Naringenin also exhibited anti-inflammatory activity and cytotoxicity in carcinoma cells [85,86].

### 2.6. Extraction of Insoluble-Bound Phenolics

Insoluble-bound phenolics, as already mentioned, are covalently bound to the cell wall matrix via ester, ether and C-C bonds, thus they should be hydrolyzed/liberated from the cell wall matrix in order to measure their contents. Acid and alkaline hydrolysis are the most common chemical methods used to extract the insoluble-bound phenolics and recently, many other new methods such as enzymatic hydrolysis and microwave assisted hydrolysis have been employed for better release of insoluble-bound phenolics from cell wall matrices.

#### 2.6.1. Acid Hydrolysis

Acid hydrolysis for the extraction of insoluble-bound phenolics from food materials has been widely employed by using 1%–5% hydrochloric acid in water/methanol. The advantages of acid hydrolysis are its convenience and simple steps for the extraction. The extracted bound phenolics can be used directly for further experimentation after neutralization and filtration, unlike alkaline extraction which requires an additional extraction procedure using diethyl ether. However, phenolic compounds are unstable at low pH, thus they can be degraded during the extraction process or storage. For example, Sani et al. [87] compared acidic and alkali hydrolysis for releasing of phenolics from germinated brown rice. Their results showed that some phenolics such as hydroxycinnamic, caffeic, syringic and protocatechuic acids were found in alkali hydrolysates, but were absent in acid hydrolysis products. This result may probably be due to the degradation or structural changes in the phenolics upon acid hydrolysis. According to the literature, flavonol extracts from *Opuntia ficus indica* prickly fruit were partially degraded during acid hydrolysis [88]. In addition, Fazary and Ju [89] argued that acid hydrolysis was not appropriate for hydrolyzing ester bonds of insoluble-bound phenolics, even though the treatment showed efficient hydrolysis in breaking the glycosidic bonds. Thus, alkali hydrolysis has been commonly used for the release of insoluble-bound phenolics from the food matrix rather than acid hydrolysis.

#### 2.6.2. Alkaline Hydrolysis

The most common chemical method for extraction of insoluble-bound phenolics is alkaline hydrolysis that uses a wide range of concentrations of sodium hydroxide. This hydrolysis method has proven to be effective in hydrolyzing both ether and ester bonds that are rarely broken by the acid hydrolysis process [90]. In addition, normally alkaline hydrolysis is conducted at room temperature, leading to low rate of loss of phenolics during the process than the acid hydrolysis method [91]. Alshikh et al. [24] studied insoluble-bound phenolics in six lentil cultivars using alkali hydrolysis and found phenolic acids such as gallic acid, protocatechuic acid, *p*-coumaric acid and ferulic acids. The alkali hydrolysis was also employed for exploring the insoluble-bound phenolics in fruit seeds [35]. In addition, alkali hydrolysis has been widely used for the release of insoluble-bound phenolics in many types of foods such as cereals, legumes and seeds [24,25,26,27,28,30]. However, the disadvantage of this method is the more complex procedure for the extraction in alkaline hydrolysis, as mentioned above, that requires further extraction steps after sodium hydroxide hydrolysis to isolate the liberated phenolics from food matrices.

#### 2.6.3. Enzymatic Hydrolysis

Aside from chemical methods such as acid and alkaline hydrolyses, enzymatic hydrolysis can serve as an efficient method for the extraction of insoluble-bound phenolics. In general, carbohydrate-hydrolyzing enzymes, including cellulase, hemicellulase, pectinase, amylase and glucanase, are used to dismantle cell wall matrices that consist of cellulose, hemicellulose, pectin and glucan [92]. The cell wall matrix disintegrated by the enzymes would expose more surface to the solvent, facilitating extraction of the insoluble-bound phenolics. The advantage of enzymatic hydrolysis extraction is the minimization of the loss of phenolic compounds due to extreme (too low or too high) pH conditions during the extraction process. Xu et al. [93] attempted the release of phenolic compounds from cell wall of muscadine grape (*Vitis rotundifolia* Michx.) skins and seeds using cellulase, pectinase and β-glucosidase. The enzymatic treatment shortened the extraction time and improved antioxidant capacity of the extracts compared to the common solvent extraction. Tang et al. [36] reported that enzymatic hydrolysis using pectinase, xylanase and feruloyl esterase released comparable amounts of bound phenolics to the soluble phenolics, but lower than acid and alkaline extraction, from quinoa seeds. Zheng et al. [94] reported that carbohydrases such as cellulases, β-glucanase and pectinase increased total phenolic contents of unripe apples, however some of the phenolics such as chlorogenic acid and phloridzin were significantly decreased during the enzymatic hydrolysis. Therefore, the enzymatic hydrolysis has both advantages and disadvantages.

### 2.7. Release of Insoluble-Bound Phenolics from Cell Wall Matrix

#### 2.7.1. Non-Thermal Processing

##### Fermentation Process in Food

Fermentation refers to the microorganism-mediated bioconversion of sugar (or monosaccharide) into lactic acid, ethanol and gas. Fermentation, as a traditional food processing method to extend the shelf-life of foods, has been suggested as an effective non-thermal food processing method for releasing insoluble-bound phenolics from the cell wall matrix of foods. During fermentation, microorganisms secrete a variety of extracellular enzymes such as carbohydrases, proteases and lipases that break down macromolecules into smaller entities so that they can use them as an energy sources and as essential ingredients. The enzymes also include cell wall matrix disintegrating enzymes such as cellulase, hemicellulase, pectinase, amylase and glucanase, leading to liberation of the insoluble-bound phenolics.

In the fermentation industry, bacteria and fungi are used for lactic acid fermentation and yeast cells are employed in ethanol fermentation. Each of the microorganisms has different enzymes, leading to individual unique flavors and final products during fermentation, driven by different enzymes that are present in the medium. For instance, representative fungi in the lactic acid fermentation industry are the *Bacillus subtilis* and *Aspergillus oryzae*. Even though, they are the same bacterial group of microorganisms, the enzymes that they secrete are obviously different. *Bacillus subtilis* releases amylase, cellulases, hydrolases, levansucrase, peptidase, proteases and xylanase, whereas *Aspergillus oryzae* releases acid protease, α-galactosidase, amylase, invertase, lignin peroxidase and tannase [95]. Therefore, the fermentation process with different species of microorganisms can result in different enzymatic reactions, followed by differentiation in the liberation of phenolics from cell wall matrix of food.

The main component of cell wall matrix is cellulose, thus cellulase plays a significant role in releasing the insoluble-bound phenolics. The hydrolysis process of cellulase can be divided into three steps. First, endoglucanase localizes at the low density of crystallinity of cellulose fiber, releasing short oligosaccharides. Second, exoglucanase and cellobiohydrolase yield cellobiose units, a disaccharide with β- linkage, from the ends. Finally, the released cellobiose is hydrolyzed by β-glucosidase into glucose units [96]. In addition, the efficiency of cellulase was improved in the presence of phenolic acids such as ferulic acid and *p*-coumaric acid which act as a cofactor and assist their enzyme activity, enhancing their activity by up to 28.3% and 15.1%, respectively [97]. Aside from cellulase, all cell wall matrix disintegrating enzymes such as cellulase, hemicellulase, pectinase, amylase and glucanase play a major role in degradation of cell walls, leading to the liberation of insoluble-bound phenolics.

Esterase also plays a significant role in the release of bound phenolics after disintegration of cell wall matrix by cellulase. They hydrolyze ester bond between phenolic acids and cell wall substances, leading to the release of phenolics. Some fungi and yeast cells such as *Lactobacillus lactis*, *Lactobacillus rhamnosus*, *Aspergilus niger*, *Cryptococcus flavus* and *Cryptococcus sp*. *S-2* release several types of esterases [95]. Representative esterases for the liberation of bound phenolics are feruloyl esterases [E.C. 3.1.1.73], which are also known as ferulic acid esterases (FAE), cinnamoyl esterases and cinnamic acid hydrolases; these are subclasses of the carboxylic acid esterases (E.C. 3.1.1.1) [37,98,99]. Tapin et al. [100] used feruloyl esterase to release phenolic compounds from wheat and flax straws and the enzyme effectively increased the liberation of phenolics such as ferulic acid, coumaric acid and vanillic acid, as well as vanillin.

Many studies have reported on the improvement of antioxidant capacity of fermented foods and have suggested that the improvement is due to the release of insoluble-bound phenolics by cell wall disintegrating enzymes. Kim et al. [101] reported that fermented rice spent water, which is the byproduct of rice milling, improved the antioxidant capacity as reflected in the DPPH radical scavenging ability and reducing power and suggested that the results might be due to the liberation of bound phenolics during fermentation. Dey and Kuhad [102] reported the antioxidant capacity of four whole grain cereals, namely wheat, brown rice, maize and oat and demonstrated that this was enhanced by solid-state fermentation using several fungi such as *Aspergillus oryzae NCIM 1212*, *Aspergillus awamori MTCC No. 548*, *Rhizopus oligosporus NCIM 1215* and *Rhizopus oryzae RCK2012*. They suggested that extracellular enzymes such as α-amylase, xylanase, β-glucosidase and esterases from fungi might play a significant role in releasing insoluble-bound phenolics from cell wall matrices of cereals, leading to increased antioxidant capacity.

##### Germination

Germination is the bioprocess that induces breaking of the dormancy of seeds by sprouting and growth. Germination is initiated with imbibition (hydration) that leads to swelling and breaking of seed coat, followed by activation of cell metabolism. The increase in water intake allows increase in the number of hydrated cells. The hydrated cell initiates aerobic respiration, mitochondrial repair, DNA repair, transcription and translation of new mRNAs at the same time, followed by DNA synthesis, cell division and seedling growth [103]. The activated cells release hydrolytic enzymes which hydrolyze macromolecules such as starch and protein into smaller molecules that are used for the metabolism and growth of the seeds, affecting the content of phenolic compounds and their formation [104].

Ti et al. [105] showed that the contents of insoluble-bound phenolics such as syringic acid, coumaric acid and ferulic acid were increased from 1.7, 34.2 and 121.6 to 6.4, 117.9 and 343.8 μg/g DW, respectively, during 5 days of germination of brown rice. Yeo and Shahidi [106] reported that the contents of insoluble-bound phenolics were enhanced during germination of lentils. The insoluble-bound phenolics of canary seeds were increased during 5 days of germination, especially ferulic acid which was the dominant bound phenolic and its content was increased from 98.20 to 313.07 μg/g DW [25]. Yang et al. [107] argued that the enhancement of insoluble-bound phenolics during germination might be due to cell division (biosynthesis) of the sprouting or growth of seeds, which increased total volume of cell wall part, followed by an increase of bound phenolics.

#### 2.7.2. Thermal and Hydrothermal Processing

##### Roasting

Roasting is a traditional method that not only improves food flavor, such as those of nuts and legumes, but also serves as a useful pretreatment means for better oil release and extraction from oilseeds [108]. During the roasting process, the heat used results in chemical reactions such as the Maillard reaction, leading to alteration of the chemical structures of phytochemicals present. In addition, the heat energy can also cause disruption of cell wall matrix of foods, enhancing the release of bound phenolics [109,110,111]. According to the literature, the roasting process affects the phenolics of nuts such as cashew nut, hazelnut and peanut, leading to alteration of their antioxidant activities [112,113]. Nyembwe et al. [114] reported that the contents of gallic acid and protocatechuic acid were increased from 34.8 and 78.0 to 81.0 and 123.0 mg/100 g DW of flour, respectively, upon roasting at 150 °C for 30 min, possibly due to the release of bound phenolics during the process. Chandrasekara and Shahidi [115] reported that the protection factor by the Rancimat assay and total phenolic content of phenolic extracts from the cashew nuts was increased by both the low and high temperature roasting treatment and discussed that the improvement can be induced by liberation of bound phenolics during the roasting process. Ee et al. [116] reported that the increase of soluble phenolics of wattle seeds after the roasting process was due to thermal dissociation of bound phenolics.

##### Extrusion Cooking

Extrusion cooking uses high temperature, pressure and shear force to make individual unique products, leading to improvement of food quality such as digestibility of starch and protein as well as enhancement of food stability due to the inhibition of enzyme activity [117]. The different characteristics of extrusion cooked foods are governed by the number of processing variables such as temperature, moisture content, pressure and processing time [118]. For instance, breakfast cereals and corn curls are made under high temperature, low moisture and high shear, while pasta is made under increased moisture content (about 40%) and low temperature, conferring specific texture and palatability. Meanwhile, the extrusion processing, particularly under high thermal and pressure conditions, influence minor components such as phenolic compounds by disrupting cell wall matrix of foods, followed by the release of insoluble-bound phenolics. For example, extrusion cooking was found to liberate bound ferulic acid from cell walls of pigmented maize [119]. Gui and Ryu [120] reported that the extrusion process enhanced total phenolic content in both the free and conjugated phenolic acids of ginsengs and this was thought to be due to the liberation of bound phenolics from the cell wall matrix. In support of these results, Ng et al. [121] reported that extrusion cooking affects cell wall matrix of onion waste, leading to depolymerization of pectins and hemicelluloses. Thus, extrusion cooking can serve as an effective means to release bound phenolics from the cell walls of foods. Meanwhile, some data indicated reduced antioxidant capacity upon extrusion processing. Altan et al. [122] reported that extrusion processing resulted in 46%–60% loss in total phenolic contents in barley compared to the corresponding raw material. The extrusion cooking reduced the antioxidant capacity of free and bound phenolics of green banana flour [118]. Flavanones and flavones of sorghum (*sorghum bicolor* L.) were significantly decreased (100%) during the extrusion cooking [123]. Thus, the negative effects indicate the need for the development of alternative extrusion conditions such as minimizing shear and temperature stress to prevent loss of bioactive compounds [124,125]. In summary, the extrusion cooking facilitates liberation of bound phenolics from cell wall matrix via hydrolysis under high temperature and pressure. On the other hand, the high energy extrusion condition degraded bioactive phenolic compounds with consequent loss of their antioxidant capacity.

Meanwhile, the baking process also affects the phenolic contents of staple foods. For example, insoluble-bound phenolics of rye whole meals were decreased upon baking and bread preparation from 1575 to 1472 µg/g dry matter; while the content of free phenolics such as ferulic acid was increased during the process due to the release of insoluble-bound phenolics during the process [126].

##### Boiling

The boiling process enhances the nutritional value, texture and acceptability of legumes as well as degrading anti-nutritional factors such as tannins that reduce the digestibility of foods by interfering with the absorption of nutrients [127]. The boiling process, which is a most common cooking method used as a hydrothermal treatment, involves heat energy, affecting the physicochemical properties of foods. The phenolics are also greatly influenced by the hydrothermal treatment that weakens the cell wall matrix and facilitates liberation of bound phenolics [128], as well as causing a variety of chemical reactions with other compounds in which changes in the content and structures of phenolics occur.

Many studies on the boiling and hydrothermal treatment of foods have shown increased content of phenolics and their antioxidant activity. The total phenolic contents of unripe and ripe plantains, eggplant and potato were increased after the boiling treatment [129,130,131]. Bellail et al. [132] investigated the effect of boiling treatment on the changes of phenolic compounds of sweet potato and showed enhanced phenolic contents in some genotypes. Antioxidant capacity of potatoes was also improved after boiling as reflected in both ABTS and DPPH results [131]. Similar studies on cooked fruits and vegetables indicated enhancement of antioxidant capacity compared to their corresponding raw materials [109,133,134].

On the contrary, some studies have revealed decreased phenolic contents after boiling treatment. Tsamo et al. [135] reported that the boiling treatment reduced total phenolic content by about 34% in peel and most of flavonols were decreased including kaempferol-3-*O*-rutinoside (61.3%) and rutin (59.8%), whereas ferulic acids were apparently increased (63.7%) in six plantain banana cultivars. Mazzeo et al. [136] reported that chlorogenic acid, quercetin and luteolin in carrots were degraded upon boiling (increase in kaempferol). Scaglioni et al. [137] investigated the effect of boiling on changes of free and bound phenolics and demonstrated that the contents of eight phenolic compounds, namely gallic, protocatechuic, chlorogenic, *p*-hydroxybenzoic, caffeic, syringic, *p*-coumaric and ferulic acids were reduced. López et al. [138] reported that the boiling treatment decreased free phenolics such as protocatechuic acid, ferulyl aldaric acid, *p*-coumaric acid, proanthocyanidin dimer, hesperetin 7-neohesperidoside, naringenin 7-rutinoside and kaempferol 3-glucoside, however digestion and absorption rate at the intestinal level were improved and their neuroprotective and anticancer activities were maintained. Siah et al. [139] studied the effect of boiling process on the phenolic profiles and antioxidant capacity of faba bean. The results showed that boiling process decreased the total contents of phenolics and flavonoids as well as the antioxidant capacity as evaluated by the DPPH, TEAC, ORAC and FRAP assays. The reduction of antioxidant capacity and phenolic content was thought to be due to the degradation of phenolics or binding with other insoluble-bound substances by the high heat energy upon boiling. Xiaoyun et al. [140] reported 3%–32% loss of ORAC value in eight potato cultivars after boiling; other studies also showed reduction of polyphenolic content upon hydrothermal treatment [141,142,143]. Hirawan et al. [144] studied on the changes of phenolic contents of pastas upon the boiling treatment and a decreased ferulic acid in six different pastas was found. Thus, the hydrothermal treatment reduces both the bound and soluble phenolics, which might be due to the chemical reaction with other compounds such as protein and forming irreversible covalent bond that is not hydrolyzed in the extraction process [145].

## 3. Conclusions

The insoluble-bound phenolics are abundant in plant-based foods, especially in the grains of cereals, pulses and oilseeds, among others. Their localization, synthesis, transfer and formation mechanism in plant cells, their metabolism in human digestive track, their bioactivity as well as release from food matrix upon food processing and extraction were found to be important as discussed in this contribution. Despite numerous reports on the contents of phenolics in natural sources and the antioxidant capacity of bound phenolics, their metabolism in biological systems and extraction and analytical methods have not been adequately established. Especially, absorption in the digestive track such as large intestine and changes of their chemical structures in the blood stream after absorption needs to be critically examined for better understanding of the metabolism of bound phenolics in biological systems.

## Figures and Tables

**Table 1:** Insoluble-bound phenolic acids in cereals, legumes and other seeds.

Sources	Bound Phenolic Acids (μg/g, DW)	Reference
Cereals		
Black rice	Protocatechuic (162.1), 4-hydroxybenzoic (21.2), vanillic (27.7), *p*-coumaric (17.6) and ferulic (64.7) acids	[20]
Maize (Pericarp)	*p*-Hydroxybenzoic (499.0), vanillic (1788.0), syringic (83.0), *p*-coumaric (3.0), ferulic (3247.0) and isoferulic (842.0) acids	[21]
Maize (Germ)	*p*-Hydroxybenzoic (21.0), vanillic (522.0), syringic (897.0), caffeic (4.0), *p*-coumaric (12.0), ferulic (247.0) and isoferulic (58.0) acids	
Maize (endosperm)	*p*-Hydroxybenzoic (10.0), vanillic (6.0), caffeic (0.6), *p*-coumaric (0.3), ferulic (27.0) and isoferulic (1.0) acids	
Durum wheat	Gallic (6.0), 4-hydroxybenzoic (4.4), vanillic (2.9), syringic (2.9), *p*-coumaric (11.8), *trans*-ferulic (216.3), sinapic (41.2) and *trans*-cinnamic (5.4) acids	[22]
Bread wheat	Gallic (5.9), 4-hydroxybenzoic (4.7), vanillic (2.9), Syringic (3.7), *p*-coumaric (13.9), *trans*-ferulic (253.1), sinapic (45.5) and *trans*-cinnamic (5.2) acids	
Barley	Gallic (5.5), protocatechuic (4.9), 4-hydroxybenzoic (5.6), vanillic (5.4), caffeic (4.6), syringic (4.6), *p*-coumaric (75.9), *trans*-ferulic (320. 8), sinapic (41.9), salicylic (9.7) and *trans*-cinnamic (5.9) acids	
Oat	4-Hydroxybenzoic (6.3), vanillic (12.9), caffeic (6.5), syringic (8.4), *p*-coumaric (603.0), *trans*-ferulic (1032.8), sinapic (100.6) and trans-cinnamic (5.4) acids	
Rye	Gallic (7.1), protocatechuic (4.2), 4-hydroxybenzoic (5.3), vanillic (4.1), caffeic (5.1), syringic (3.4), *p*-coumaric (20.7), *trans*-ferulic (216.7), sinapic (44.7) and *trans*-cinnamic (4.6) acids	
Rice	Gallic (5.1), 4-hydroxybenzoic (4.5), vanillic (1.7), syringic (2.8), *p*-coumaric (20.9), *trans*-ferulic (65.5), sinapic (22.8) and *trans*-cinnamic (4.9) acids	
Triticale	Gallic (4.8), protocatechuic (3.8), 4-hydroxybenzoic (5.4), vanillic (5.4), caffeic (4.3), syringic (4.1), *p*-coumaric (19.9), *trans*-ferulic (334.1), sinapic (58.4), salicylic (10.9) and *trans*-cinnamic (5.6) acids	
Corn	4-Hydroxybenzoic (4.7), vanillic (4.9), caffeic (4.9), syringic (4.2), *p*-coumaric (95.4), *trans*-ferulic (954.4), sinapic (78.8) and *trans*-cinnamic (5.2) acids	
Millet (7 cultivars)	Ferulic (178.0-1685.0) and *p*-coumaric (20.0-1139.0) acids	[23]
Legumes		
Lentils (6 cultivars)	Gallic acid (0.0-1.8), protocatechuic acid (0.0-4.4), *p*-coumaric acid (0.0-3.1) and ferulic acid (0.0-0.3) acids	[24]
Cranberry beans (7 cultivars)	*p*-Hydroxybenzoic (4.4-71.2), *p*-coumaric (4.9-18.4), ferulic (11.0-41.7) and sinapic (0.03-3.55) acids	[25]
Soy isoflavone concentrate	*p*-Hydroxybenzoic (12.0), vanillic (62.0), syringic (262.0), *p*-coumaric (61.0), ferulic (55.0) and sinapic (25.0) acids	[26]
Mung bean	Gallic (3.0), caffeic (0.3), *p*-coumaric (0.2), ferulic (2.2) and sinapic (1.5) acid	[27]
Pinto bean	Protocatechuic (25.6), *p*-hydroxybenzoic (42.7), *p*-coumaric (26.8), ferulic (34.7) and sinapic (89.9) acid	[28]
Black bean	Gallic (41.1), protocatechuic (14.1), *p*-hydroxybenzoic (5.6), caffeic (17.5), syringic (17.1), *p*-coumaric (22.1), ferulic (170.1) and sinapic (50.5) acid	
Kidney bean	Protocatechuic (64.4), *p*-hydroxybenzoic (21.3), *p*-coumaric (7.9), ferulic (138.0) and sinapic (73.4) acid	
Cowpea	Gallic (5.4) acid	[29]
Chickpea	Gallic (82.8) and protocatechuic (110.9) acid	[30]
Cow gram	Protocatechuic (172.6) acid	
Flower waist bean	Protocatechuic (128.4) acid	
Hyacinth bean	Protocatechuic (111.8) acid	
Pearl bean	Protocatechuic (121.9) acid	
Oilseeds		
Sunflower	Gallic (11.2), protocatechuic (50.8), caffeic (25.5), *p*-coumaric (2.5), ferulic (16.9) and sinapic (15.6) acids	[27]
Rapeseed meal (seven cultivars)	389-1050 μg sinapic acid equivalent/g DW (Main phenoic acids: sinapic, *p*-coumaric and *trans*-ferulic acid)	[31]
Flaxseed	*p*-Coumaric acid glucoside (3800.0) and ferulic acid glucoside (4400.0)	[32]
*Moringa oleifera* seed flour	Gallic (15.9) and *p*-coumaric (7.4) acids	[33]
Soy bean	Gallic (64.2), protocatechuic (238.8), vanillic (88.7), syringic (121.6), *p*-coumaric (101.7) and ferulic (87.1) acid	[34]
Fruit seeds		
Blackberry seed meal	Protocatechuic (2.2), *p*-coumaric (0.2), gallic (3.4) and caffeic (2.1) acids	[35]
Black raspberry seed meal	Protocatechuic (0.9), *p*-coumaric (2.4), gallic (2.4) and caffeic (1.5) acids	
Blueberry seed meal	Protocatechuic (0.3), *p*-coumaric (0.2), gallic(3.5) and caffeic (0.2), syringic (0.9) acids; gallic hexoside (1.0)	

**Table 2:** Insoluble-bound flavonoids in cereals, legumes and other seeds.

Sources	Bound Flavonoids (μg/g, DW)	Reference
Cereals		
Maize (pericarp)	Kaempferol (15.9)	[21]
Maize (germ)	Quercetin (28.8)	
Quinoa seed	Catechin (16.28), quercetin (47.2) and kaempferol (30.4)	[36]
Dent corn (pericarp portion)	Quercetin (35.9)	[37]
Flint corn (germ portion)	Quercetin (12.0)	
Rice	240 μg catechin equivalent/g DW	[34]
Corn	760 μg catechin equivalent/g DW	
Wheat	430 μg catechin equivalent/g DW	
Barley	370 μg catechin equivalent/g DW	
Legumes		
Lentils (6 cultivars)	Catechin (15.0–78.4), epicatechin (0.5–7.9), (+)-catechin-3-glucoside (0.0–122.0) and luteolin 3′-7-diglucoside (0.0–49.3)	[24]
Cranberry beans (4 cultivars)	Kaempferol (0.2–0.3)	[25]
Mung bean	Quercetin (0.2) and kaempferol (0.1)	[27]
Black bean	(+)-Catechin (109.7), epicatechin (93.8), rutin (93.8), isoquercitrin (462.3) and quercetin (86.6)	[30]
Cow gram	Quercitrin (105.4)	
Hyacinth bean	Quercitrin (91.9)	
Pearl bean	(+)-Catechin (100.7)	
Red bean	Rutin (89.5)	
Red kidney bean	Catechin (88.2) and isoquercitrin (97.1)	
Spring bay bean	Catechin (158.8), rutin (124.2), isoquercitrin (85.7) and quercetin (86.8)	
Oilseeds		
*Moringa oleifera* seed flour	Catechin (7490.0), epicatechin (810.0) and quercetin (18.7)	[33]
Sunflower	Quercetin (1.5), kaempferol (0.5) and apigenin (2.9)	[27]
Soy bean	Epicatechin (95.2), isoquercitrin (396.0) and quercetin (101.0)	[30]
Fruit seeds		
Blackberry	(+)-Catechin (1.0), (−)-epicatechin (1.1), quercetin (2.0), quercetin pentose (3.3), epicatechin gallate (1.1) and quercetin-3-*O*-glucuronide (5.1)	[29]
Black raspberry	(+)-Catechin (0.4), (−)-epicatechin (3.1), quercetin (3.2), epigallocatechin (1.3), epicatechin gallate (0.9) and quercetin-3-*O*-glucuronide (11.4)	
Blueberry	(+)-Catechin (0.7), (−)-epicatechin (0.07), quercetin (0.2), epigallocatechin (0.4), myricetin (0.2), quercetin pentose (0.5) and kaempferol hexoside (0.6)	
